# Whole transcriptomic network analysis using Co-expression Differential Network Analysis (CoDiNA)

**DOI:** 10.1371/journal.pone.0240523

**Published:** 2020-10-15

**Authors:** Deisy Morselli Gysi, Tiago de Miranda Fragoso, Fatemeh Zebardast, Wesley Bertoli, Volker Busskamp, Eivind Almaas, Katja Nowick

**Affiliations:** 1 Department of Computer Science, Leipzig University, Leipzig, Germany; 2 Fundação Cesgranrio, Rio de Janeiro, Brazil; 3 Department of Biology, Chemistry, Pharmacy, Freie Universitaet Berlin, Berlin, Germany; 4 Department of Statistics, Federal University of Technology - Paraná, Curitiba, Brazil; 5 Center for Regenerative Therapies (CRTD), Technical University Dresden, Dresden, Germany; 6 Dept. of Ophthalmology, Universitäts-Augenklinik Bonn, University of Bonn, Bonn, Germany; 7 Department of Biotechnology, NTNU - Norwegian University of Science and Technology, Trondheim, Norway; 8 K.G. Jebsen Centre for Genetic Epidemiology, NTNU - Norwegian University of Science and Technology, Trondheim, Norway; Universidad de Jaen, SPAIN

## Abstract

Biological and medical sciences are increasingly acknowledging the significance of gene co-expression-networks for investigating complex-systems, phenotypes or diseases. Typically, complex phenotypes are investigated under varying conditions. While approaches for comparing nodes and links in two networks exist, almost no methods for the comparison of multiple networks are available and—to best of our knowledge—no comparative method allows for whole transcriptomic network analysis. However, it is the aim of many studies to compare networks of different conditions, for example, tissues, diseases, treatments, time points, or species. Here we present a method for the systematic comparison of an unlimited number of networks, with unlimited number of transcripts: **Co**-expression **Di**fferential **N**etwork **A**nalysis (CoDiNA). In particular, CoDiNA detects links **and** nodes that are common, specific or different among the networks. We developed a statistical framework to normalize between these different categories of common or changed network links and nodes, resulting in a comprehensive network analysis method, more sophisticated than simply comparing the presence or absence of network nodes. Applying CoDiNA to a neurogenesis study we identified candidate genes involved in neuronal differentiation. We experimentally validated one candidate, demonstrating that its overexpression resulted in a significant disturbance in the underlying gene regulatory network of neurogenesis. Using clinical studies, we compared whole transcriptome co-expression networks from individuals with or without HIV and active tuberculosis (TB) and detected signature genes specific to HIV. Furthermore, analyzing multiple cancer transcription factor (TF) networks, we identified common and distinct features for particular cancer types. These CoDiNA applications demonstrate the successful detection of genes associated with specific phenotypes. Moreover, CoDiNA can also be used for comparing other types of undirected networks, for example, metabolic, protein-protein interaction, ecological and psychometric networks. CoDiNA is publicly available as an R package in CRAN (https://CRAN.R-project.org/package=CoDiNA).

## Introduction

Complex systems, exemplified by biological pathways, social interactions, and financial markets, can be expressed and analyzed as systems of multi-component interactions [1, 2]. In systems biology, it is necessary to develop a thorough understanding of the interactions between factors, such as genes or proteins. Gene co-expression networks have been especially effective in identifying those interactions [3–6]. In gene co-expression networks, nodes represent genes and a (*weighted*) link between a pair of genes represents their connection; often calculated from a similarity measure such as correlation or mutual information [7–9]. The sign of the correlation may suggest an up- or down-regulation of one factor by the other [8, 10, 11]. It has been shown that different conditions have distinct underlying regulatory patterns and therefore will lead to dissimilar networks even for a single system [1, 8, 12, 13] and genes that are co-expressed are more likely to act as regulators [10, 14]. Similar systems also lead to different networks, due to technical or biological background noise. This type of inconsistency can be solved by combining multiple similar networks into a Consensus Network [8]. While the analysis of expression differences allows for identification of genes that are significantly differentially expressed among two or more conditions [5, 15–18], it does not enable the investigation of more complex patterns, such as changes in the rewiring of the regulatory relationships of genes [19, 20].

Differential network analyses are able to capture changes in gene relationships and are thus exceptionally suitable for understanding complex phenotypes and diseases [5]. Several methods for **pairwise** network comparisons exist, for example: CoXpress [21], CSD [22], DCGL [20, 23], DICER [14], DiffCoEx [24], DiffCorr [15, 25], Gain [26], MIMO [27], ModMap [28], NetAlign [29], SAGA [30], the discordant method [31] and QNet [32]. In Table 1 we present a brief comparison of those methods along with the ones that compare more than two networks. Each of those methods relies on different input data to either construct and later compare or solely compare the networks. Extensive comparisons of similarities and differences in these methods can be found elsewhere [10, 18, 19, 22, 33].

**Table 1:** Methods for comparing co-expression networks: The columns inform about the Number of networks that can be compared; Statistical methodology used; Whether the focus of comparison is on nodes or links.; Output type; if a Visualization tool is integrated; and Availability of the method.

Method	Ref.	W	Description	Nodes or Links	Output	Visual tool	Available	Network Construction	Network size
**CoDiNA**		> = 2	Geometrical transformation, Normalized scores for links and classification of nodes.	Links and nodes	Full network, Nodes and Links classification	Yes	R package	No	Small, medium and large
**CompNet**	[1]	> = 2	Jaccard-similarities from the union, intersections and exclusive links.	Links	Full network	Yes	GUI *	No	Small and medium
**ConMOd**	[34]	>2	Finds conserved functional modules across multiple biological networks by transforming multiple networks into two feature matrices, factorizing the two feature matrices into consensus factors and a soft node selection	Links and nodes	Conserved modules in multiple networks	No	MATLAB	No	Small and medium
**CoXpress**	[21]	2	Hierarchical cluster analysis on the expression values	Nodes	Cluster of genes for hierarchical each group	Yes	R package	Gene modules	
**CSD**	[22]	2	Score the links to construct a unified differential co-expression network	Links	Full network	No	In-house software **	Yes	Small, medium and large
**DCEA**	[35]	2	Calculates the expression correlation changes of gene pairs between two conditions. Characteries a node condition by comparing the numbers of gene neighbors in different coexpression networks.	Links and nodes	Differential Regulated Links involved differential co-expression links and transcriptional regulation links	No	R package	Yes	
**DCGL**	[20, 23]	2	Highlights a subset of differentially co-expressed genes and links as either differentially regulated genes or differentially regulated links	Links and nodes	Differential Regulated Links involved differential co-expression links and transcriptional regulation links	Yes	R package	Yes	Small, medium and large
**DICER**	[14]	2	Probabilistic score for differential correlation	Nodes	Cluster of genes in each module	No	GUI *	Yes	
**DiffCoEx**	[24]	> = 2	Identifies gene coexpression differences between multiple conditions based on WGCNA modules	Nodes	Cluster of genes in each module	No	In-house software **	Gene modules	
**DiffCorr**	[15, 25]	2	Fisher’s z-test	Links	Full network	Yes	R package	Yes	Small and medium
**Discordant Method**	[31]	2	Categorizes the correlation types for each group using Fisher’s transformation and later uses the concordant method for microarrays	Links	Specific links for each category	No	R package	Yes	
**ECF-Statistic**	[37]	2	Uses a conditional F-Statistic to calculate differences in co-expression	Links and nodes	Genes that are differentially co-expressed in each sample	No	In-house software **	Differential gene-gene co-expression patterns	
**Gain**	[26]	2	Calculates the Jaccard, Simpson, Geometric, Hypergeometric and Cosine indexes and Pearson correlation for links.	Links	Full network	Yes	Web-based	Yes	Small
**GNAT**	[36]	> = 2	Using a hierarchical data, it finds genes that are specific for each “branch”	Nodes	Conserved and specific modules in each hierarchical	No	Web-based	Yes	
**MIMO**	[27]	2	Sub-graph matching	Nodes	Sub-graph	No	In-house	No	software **
**ModMap**	[28]	2	Identifies modules of differential genes. Based on unweigheted networks	Nodes	Cluster of genes in each module	No	In-house software **	Both	Small and medium
**NetAlign**	[29]	2	Identifies conserved structures from topology and sequence similarity	Nodes	Conserved Network Structures	No	Web-based	No	
**QNet**	[32]	2	Computes graph similarities from trees for the nodes based on colouring graph theory	Nodes	Full network	No	In-house software **	No	
**SAGA**	[30]	2	Computes graph similarities for the nodes	Nodes	Node gaps, node mismatches and graph structural differences	No	Web-based	No	Small
**TAN**	[38]	> = 2	Defines a “tissue-specific” network. Based on the average expression defines tissue specific genes	Links and nodes	Tissue specific genes and its networks	No	In-house software **	Yes	Small, medium and large

It is often aimed for a comparison of more than two networks simultaneously, such as gene co-expression networks arising from different species, tissues or diseases, or co-existence networks from different environments. Existing methods for contrasting multiple networks focus on identifying modules of differentially co-expressed genes [1, 20, 23, 34–36], thereby allowing the identification of gene groups that are functionally related. Note that, such module-centric analyses do not enable a straightforward identification of which *links* have changed between networks or which *nodes* are most differentially connected in the networks. Moreover, by focusing on subnetworks, modules, communities or clusters instead of whole transcriptome networks, scientific insight might be limited. To address these shortcomings, we present here a method that compares multiple networks of unlimited size at the level of links and nodes.

Our novel method, CoDiNA (**Co**-expression **Di**fferencial **N**etwork **A**nalysis), is implemented as an R package that also includes an interactive tool for network visualization. Our method was first employed to find common, specific or different links and nodes in a study of neurogenesis of induced Pluripotent Stem Cells (iPSC) with or without the presence of the micro RNA (miR) − 124 [13]. There, CoDiNA identified links that existed specifically only in the miR − 124 knockout or wildtype cells. A TF with yet unknown functions, ZNF787, seemed to be one important determinant for changes at the time point of highest network differentiation. Overexpressing ZNF787 during neurogenesis resulted in explicit repression of neuronal differentiation. The power and versatility of the CoDiNA method are demonstrated here using two example applications comparing more than two networks. We compare expression data from active tuberculosis (TB) patients with or without HIV. The co-infection of HIV and TB is of strong medical importance whereas, to this date, it is challenging to detect TB in the presence of an HIV infection. In another application, we compare three glioma cancer types to understand commonalities and differences in their molecular signatures.

### Existing methods for comparing more than two networks

There are important conceptual differences between our proposed method, CoDiNA, and other methods for multiple network comparisons (Table 1). Those differences do not allow for a quantitative comparison of all the methods. Most of the methods have a limitation for network size. Many methods construct their own networks, preventing the researcher to decide which network construction method is most appropriate for the available data. Nevertheless, we make a descriptive comparison, so that the researcher can decide on the best and most adequate method for their study aim.

Kuntal et al. [1] proposed a method, CompNet, that address the comparison of multiple small and medium sized networks. However, the focus of CompNet is on the visualization of the union, intersections and exclusive links of the analyzed networks. ConMOd [34] has recently been developed to find conserved functional modules across multiple biological networks. Another method, DCEA [35], measures the average co-expression difference in each gene, resulting in Differentially Coexpressed Genes, and subsequently infers the enrichment of links for each gene (Differentially Coexpressed Links). The Expected Conditional F-statistic (ECF-statistic) can also be used to compare multiple groups of co-expressed genes. This method identifies gene interactions (links) that are common or different in multiple networks [37]. DCGL [20, 23] finds genes where the variance is small across different conditions, removes them, filters for links using a *half-threshold* and follows with the network construction in order to define modules of gene-interactions. They implemented five methods for finding the differential co-expression modules. GNAT (Gene Network Analysis Tool) [36] is a method that allows comparing multiple tissues. Using a hierarchical system that encodes for tissue similarity, GNAT was used to compare the co-expression networks of 35 human tissues. Because this method assumes a hierarchy, it is necessary that the analyzed networks are related. Another method focused on finding differences on tissue co-expression in Tissue Aggregated Networks (TAN) [38]. Most of those methods focus on finding modules of differentially co-expressed genes, rather than on pinpointing conserved or changed links as CoDiNA does. Of the existing method we chose CompNet as the most established method that is able to compare more than two networks without performing its own network construction for an analytical comparison to CoDiNA. This comparison is presented after describing the results of CoDiNA for two new use cases.

## Results

### CoDiNA: An overview

**Fig 1:**
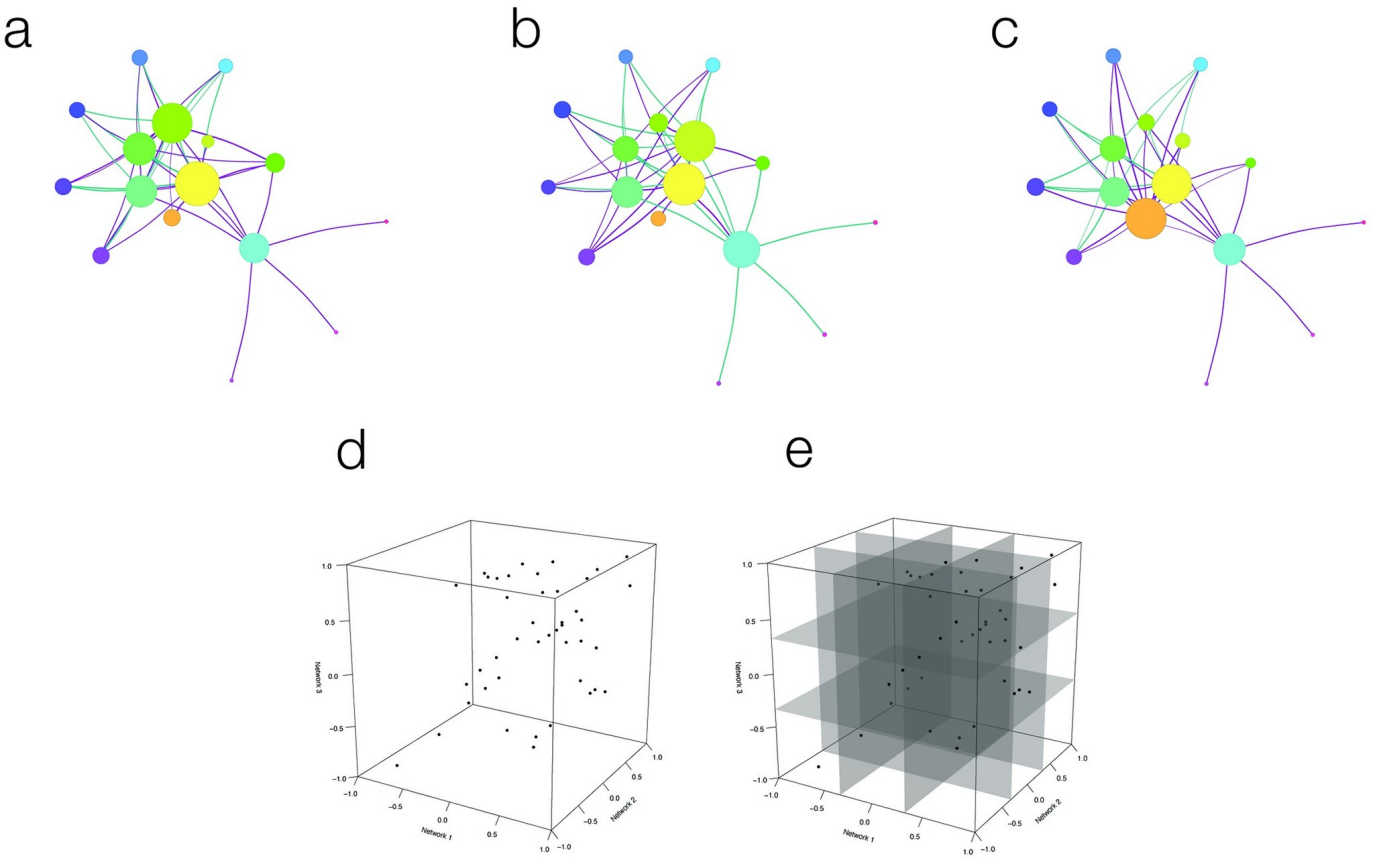
Visual representation of the CoDiNA method for a 3-network comparison. **1a**, **1b** and **1c** display three independent networks to be compared; violet links represent positively correlated gene-pairs, and green links negatively correlated ones. Node-size is relative to node strength. **1d** shows the geometrical representation of CoDiNA: a 3D scatter-plot that is derived from plotting the weights of each link in the three networks. **1e** displays the cube *segments* based on the *τ*-threshold.

CoDiNA requires as input a set of independently constructed undirected networks to be compared (Fig 1a, 1b and 1c). These could be any kind of undirected—weighted or unweighted—networks (e.g., protein-protein interaction networks, metagenomics networks, co-occurrence networks etc.); however we focus here on co-expression networks. The CoDiNA method can be divided into five steps, which will be described in detail in the Materials and Methods section:

1. Remove nodes that were **not measured** in all networks to be compared;
2. Define a minimum cutoff for the weight a link needs to have to be considered present;
3. Remove links that are *absent* in all network;
4. Classify (and sub-classify) and score links as common, specific or different between networks; denoted as Φ and $\tilde{\Phi }$, respectively;
5. Classify the nodes.

The input networks for the comparison can be constructed using any correlation method (such as Pearson or Spearman correlations, wTO [8], WGCNA [9]), or can also be Consensus Networks (CN) derived from similar networks to achieve higher confidence in the network inference [8]. In each network, the weight value of the link between the genes *i* and *j*, denoted by *ρ*_*i*,*j*_, is defined within the interval [−1, 1] (Fig 1d). To denote links as positive, negative, or neutral, this interval is divided into three parts (Fig 1e) determined by the threshold value *τ*, which by default is set to be *τ* = 1/3.

Caution is necessary when claiming that a particular gene is associated with a specific condition, since technical reasons might have precluded measuring the expression of that gene in other conditions. In other words, if no measurement exists for a gene, we have no information about its expression level—it might be not expressed or it might not have been measured. In order to avoid false associations, i.e. the incorrect inference that a particular gene is associated with a specific condition, all investigated **nodes** should be present in **all** networks. There are other experimental setups, where we might know that a gene indeed does not exist in a certain condition. For instance, when comparing networks between species, we might know based on phylogenetic analyses that a gene is indeed species specific. Further, a gene might also be non-existent due to a knock-out in an experimental condition. In the methodology section, we will give a detailed description of how we deal with nodes missing from some networks to avoid losing the information about genes in the analysis.

**Fig 2:**
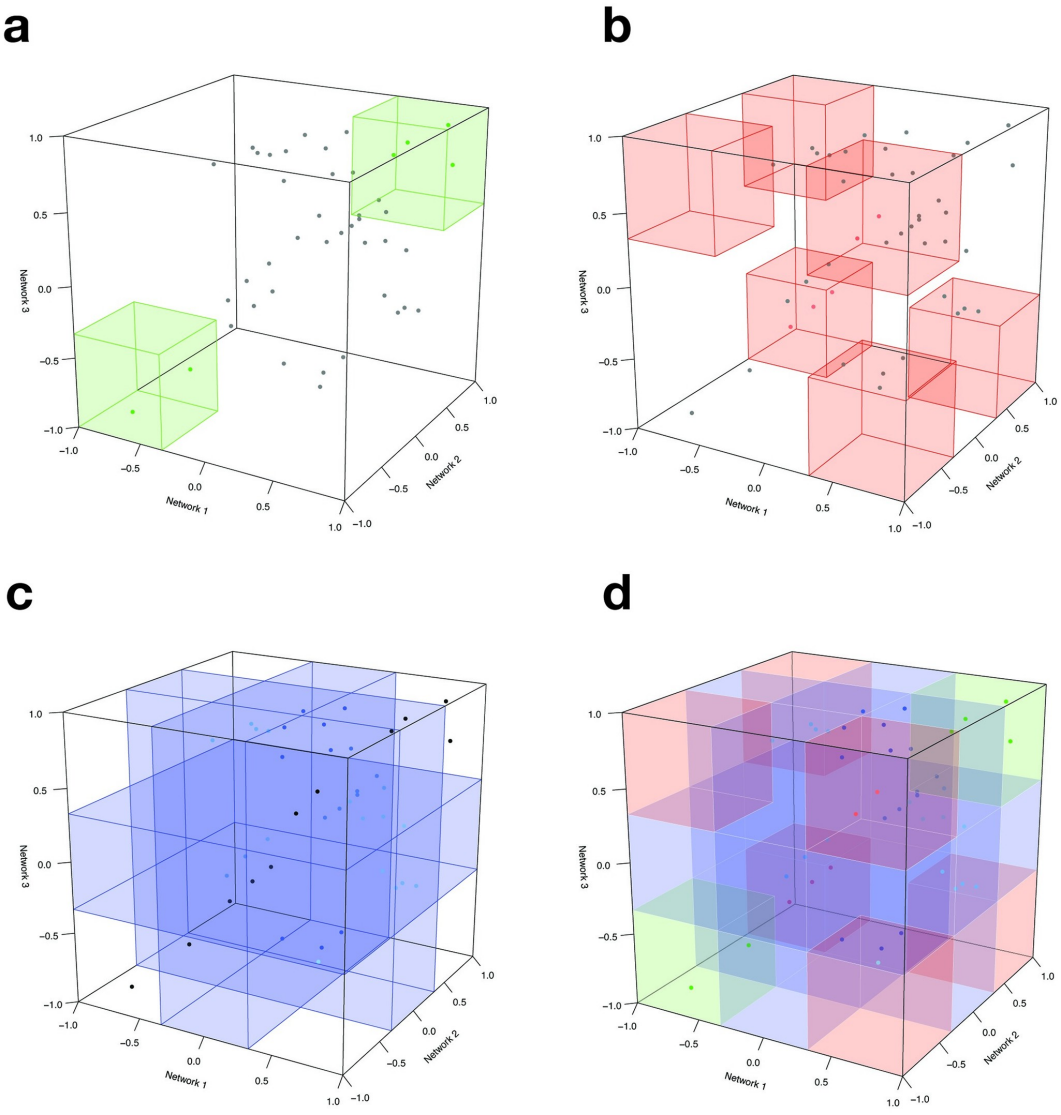
Visual representation of the CoDiNA method for a 3-network comparison: Categories definition. **2a** represents where the *α* links lie in the 3D space. **2b** and **2c** represent the locations of *β* and *γ* links, respectively. The complete set of Φ and $\tilde{\Phi }$ positions is shown in **2d**.

The output of said comparison will be a classification of each link in terms of its presence and interaction throughout the compared networks. The links are defined to belong to one out of three categories, namely:

- Common: One link is said to be an *α*-link (or *α* for short) if it is present in all networks with the same sign, i.e., it is an interaction that is **common** to all networks. If such an interaction was changed, this might happen at high costs for the organism, potentially leading to disease states (Fig 2a);
- Different: Conversely, a link is defined to be a *β*-link (or *β* for the sake of brevity) if it is present in all networks but with **different** signs of the link’s weight, i.e., it represents a different kind of interaction in at least one network. The biological interpretation of this category is that a particular gene changed its function so that a gene that up-regulates another gene in one condition down-regulates the same gene in another condition (or vice-versa), and this change might lead to a disease state (Fig 2d);
- Specific: All other links are then said to be *γ*-links and referred simply as *γ* for the remainder of this text. Those links are present in some networks but **not all**, regardless of the sign of the link’s weight, i.e., this link is specific to at least one network. This category identifies rewiring of the network topology, meaning that genes can regulate each other (or not) depending on the condition, e.g., when changing metabolic pathways (Fig 2c).

The link taxonomy induces a full network structure in which every link is classified as *α*, *β* or *γ*. To further characterize *how* a particular link is different or specific, we assign a subcategory, $\tilde{\Phi }$ (Fig 2d). This subcategory aggregates information of the Φ by clarifying to which network a link is specific or in which condition it has changed.

**Fig 3:**
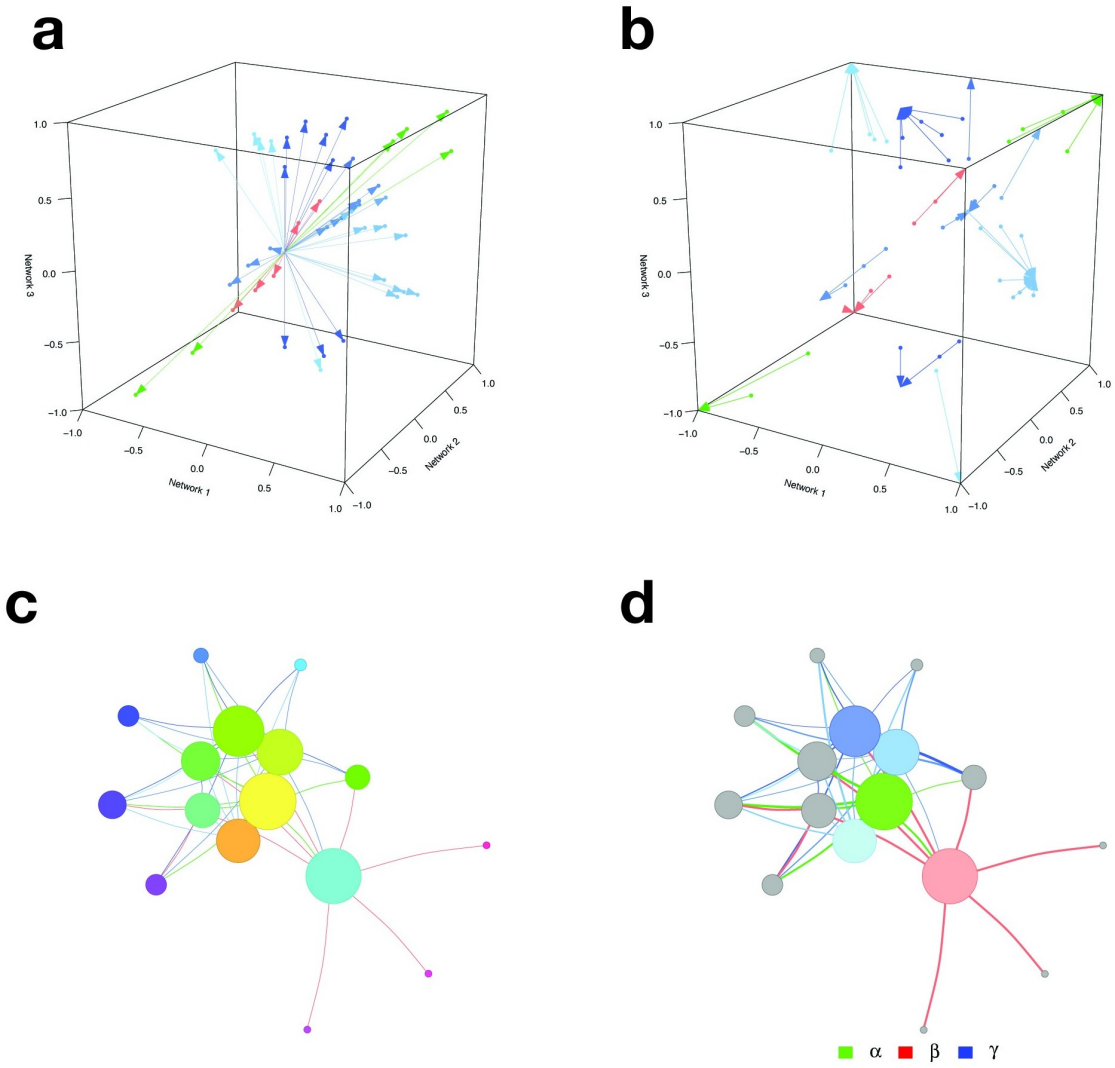
Visual representation of the CoDiNA method for a 3-network comparison: Scores definition. The *strength score* ($\Delta_{i,j}^{*}$) and the *internal score* ($\Delta_{{\tilde{\rho }}_{i,j}})$ are shown in panels **3a** and **3b**, respectively. The $\Delta_{i,j}^{*}$ score is calculated as the Euclidean distance from the center of the cube to the set of links. This score allows the selection of strong links. Links that have a big variation in their weight have lower scores, while links with higher similarities have higher scores. The second score, $\Delta_{{\tilde{\rho }}_{i,j}}$, is the distance from the link weight to the categorical weight (${\tilde{\rho }}_{i,j}$) and allows the selection of links that were well-classified in the Φ category. Links with a low $\Delta_{{\tilde{\rho }}_{i,j}}$ score are not assigned to a particular Φ category, while well-classified links receive higher $\Delta_{{\tilde{\rho }}_{i,j}}$ scores. Both scores can be combined to visualize the filtered network that contains only strong and well-classified links, as shown in **3c**. Finally, the CoDiNA network with classified links and nodes is displayed in panel **3d**.

After all links are classified, they receive two scores, a strength score ($\Delta_{i,j}^{*}$) and a internal score ($\Delta_{{\tilde{\rho }}_{i,j}}$), where *i* and *j* are the node indices. These scores are used to filter the networks for background noise (Fig 3a and 3b). One score measures the distance of a link regarding its weight measures in all networks and the other measures how well classified in the Φ category a link is. The ratio of those scores enables a filter on the network that corrects for background noise (Fig 3c).

Finally, the nodes are classified. Nodes are defined as *α*, *β*, or *γ*, respectively, if they have a significant number of links of a particular Φ and $\tilde{\Phi }$ category (Fig 3d). This node classification allows identifying nodes that are very conserved or have strongly changed in their set of links between networks and enables further functional characterization of network similarity and differences, by performing Gene Ontology Enrichment analyses.

Further details of CoDiNA are given in the Materials and Methods section.

### Running time

**Fig 4:**
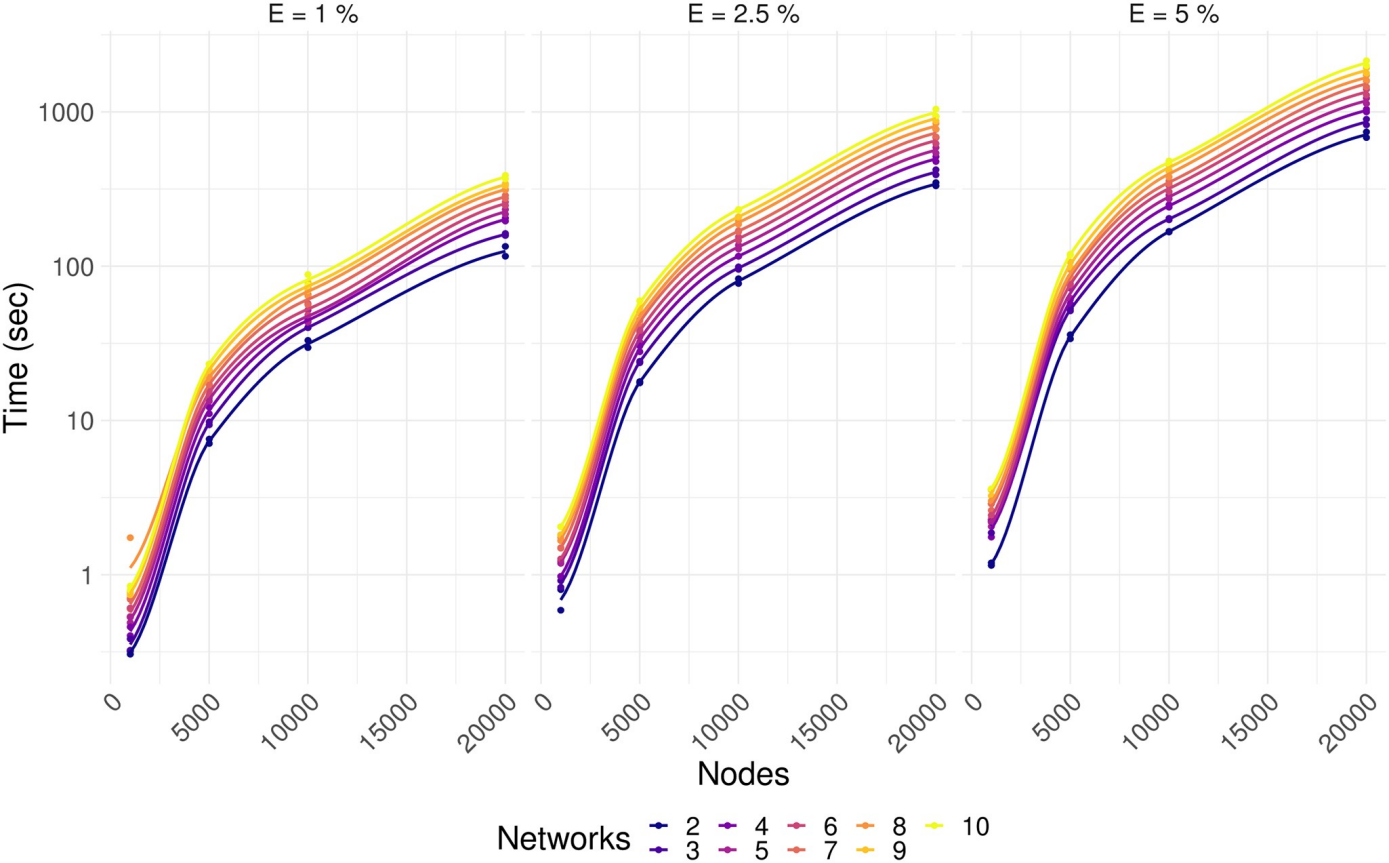
Running time for CoDiNA depends log linearly on the number of nodes, percentage of completeness of a network, and the number of networks under comparison.

The running time of CoDiNA depends on the number of nodes in the networks, the number of links in each network, and the number of networks being compared (Fig 4). To provide information about the duration of CoDiNA analyses, we simulated the comparison of 2 to 10 networks with 1000, 5000, 10000 and 20000 nodes, with different percentages of links, 1%, 2.5% and 5%. Since gene co-expression networks have on average 1% of completeness [39], and protein-protein interaction networks have on average 0.2% completeness [13], our simulations with 5% of completeness are exaggerations compared to what is expected in a natural occurring network. Networks were simulated using an Erdős-Rényi model, in a standard notebook.

### Pairwise version of CoDiNA detects network changes in induced pluripotent stem cells undergoing neurogenesis

A pairwise network comparison was already successfully applied to an expression dataset of human induced Pluripotent Stem Cells (iPSC) [13]. The iPSCs were induced to undergo neuronal differentiation within four days. We compared expression patterns of wildtype and miR-124 knock-out iPSCs over the time course of differentiation with the goal to uncover the function of miR-124 during neurogenesis. The experiment was conducted in seven replicates to facilitate the construction of a co-expression network for each day for the wildtype and the knockout cells. Using the pairwise version of CoDiNA, we revealed strong network differences between wildtype and knockout cells on day 3 of neuronal differentiation. For that day, CoDiNA classified the transcription factor (TF) ZNF787 as the TF with most specific links, suggesting it as one of the drivers of miR-124 induced network changes. Other TFs that CoDiNA identified as important in day three of neurogenenis and that also can affect the cascade were TAF2, RBM5, MAFA, ZCCHC11, BPTF, ZNF611, LCOR, PSMC3, SNRPB and CDKN1A. Since ZNF787 is more highly expressed in miR-124 knock-out compared to wildtype iPSC, we overexpressed ZNF787 in the wildtype cells to experimentally validate our result. This overexpression resulted in neuronal differentiation, however, with clear alterations in expression of genes with neuronal functions and associated with ZNF787 in the CoDiNA network. These results strongly suggest ZNF787 as one repressor of neuronal features and demonstrated that predictions found by CoDiNA could be experimentally validated. Future work can still validate the role of the other candidates during neurogenesis.

### Applications of CoDiNA comparing more than two networks

Here we present two new examples of applications of the CoDiNA method to compare multiple networks. In the first example, we use CoDiNA to analyze two tuberculosis (TB) studies with patients with and without human immunodeficiency virus (HIV) infection. The second showcase uses data from a study with patients with three types of glioma.

#### CoDiNA identified signatures of HIV in children and adults with or without tuberculosis

We used publicly available data to investigate how the transcriptome of patients and adults with TB infections can be modified by the presence of HIV. The datasets contain expression data from peripheral blood of children and adults from two TB and HIV studies. In this application, our aim is to identify similarities and differences in TB and HIV in both age groups. Both studies are available at GEO; the first one (GSE39941 [40]) contains gene expression data from 192 children with TB from Kenya, South Africa and from Malawi (HIV^+^ *n* = 69, HIV^−^ *n* = 123); the second one (GSE37250 [41]) contains expression data from 197 adults with TB from South Africa and Malawi (HIV^+^ *n* = 99, HIV^−^ *n* = 98). Both studies aimed to define transcriptional signatures for detection of TB in patients with and without HIV. We used the raw data provided at GEO [42, 43], pre-processed and normalized them and performed quality control using the R package lumi [44–46].

Networks were generated separately for adults and children that are HIV^+^ or HIV^−^. For robust network inference, we used the weighted Topological Overlap (wTO) method to generate these networks [8]. This method allows for the distinction of positive and negative interactions and corrects for background noise, allowing the network to have a lower false positive rate [8, 11]. Using the wTO R package [8, 47], it also assigns p-values to each link [8], reducing even more the number of false positive links to not confound the differential network analysis. We ran the R package wTO [8, 47] with Pearson product-moment correlation coefficient for all the 13, 817 genes with 1, 000 bootstraps and set wTO values (*ω*_*i*,*j*_) that were not significant (BH adjusted *p*-value ⩾ 0.001) to zero. The filter was used to remove false positive interactions from the final networks. Finding a large absolute *ω* for a pair of genes means that the expression patterns of both genes are strongly (positively or negatively) correlated.

**Fig 5:**
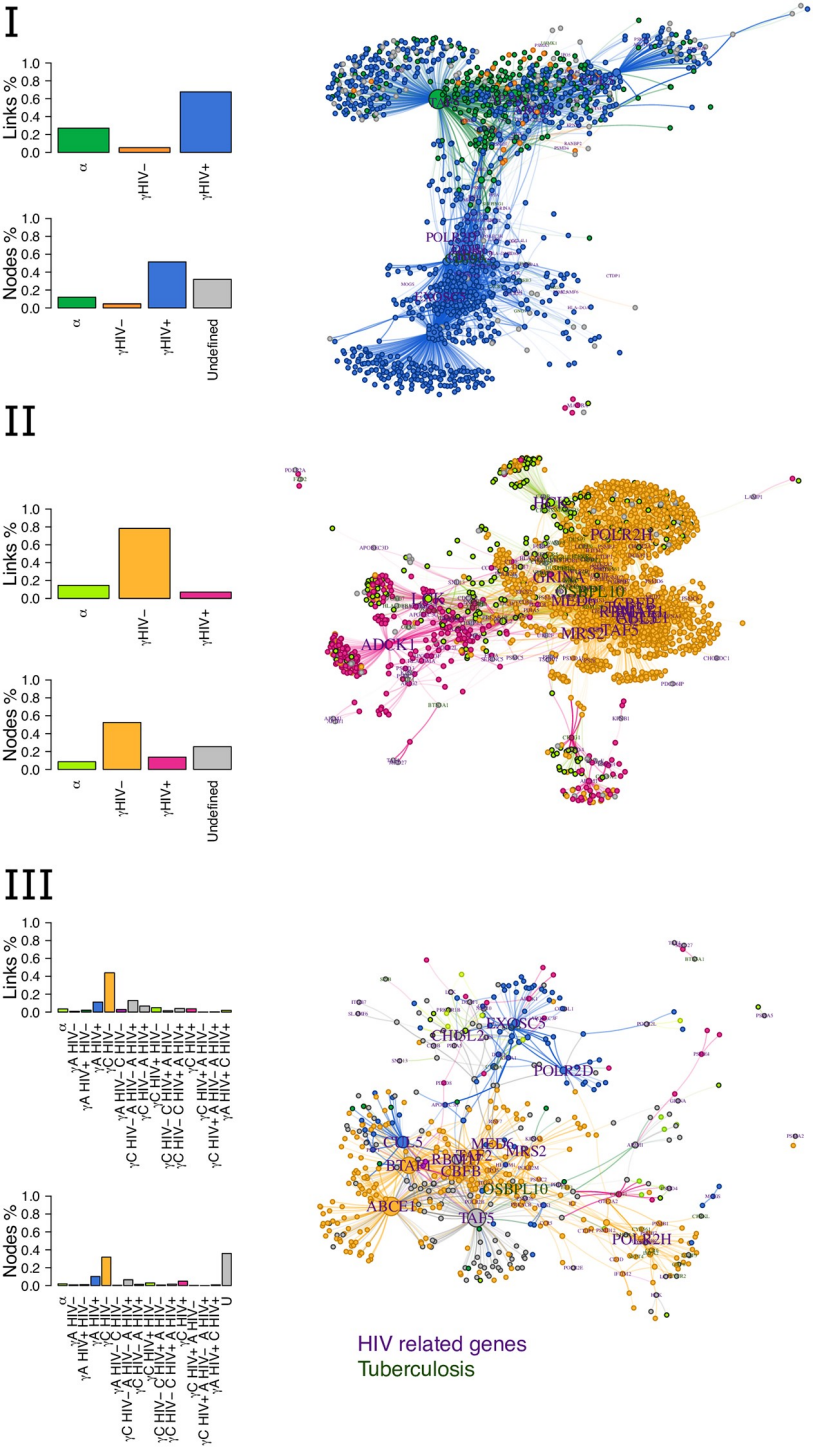
**Comparing children and adults with tuberculosis and tested for HIV:** The three panels show the categories of links and nodes for **Panel I** the CoDiNA network comparing adults with our without HIV; **Panel II** the CoDiNA network comparing children with or without HIV; and **Panel III** the complete CoDiNA network including adults (A) and children (C) with or without HIV. Note that, in the adults network (**Panel I**) most links are specific to HIV^+^ state, while for children (**Panel II**), most links are specific to individuals without HIV. Judging from **Panel III**, many links are lost in adults and in HIV^+^ children compared to HIV^−^ children.

The resulting four wTO networks were then compared using CoDiNA (Fig 5). We performed three comparisons: (i) For adults with TB we compared HIV^−^ vs HIV^+^; (ii) for children with TB we compared HIV^−^ vs HIV^+^; (iii) and we compared adults and children with TB with HIV^−^ and HIV^+^. In order to associate the $\tilde{\Phi }$ links to each one of the genes and to select the strongest and well-classified links, we used the CoDiNA networks filtered for values where the ratio of the $\Delta_{i,j}^{**}$ and $\Delta_{{\tilde{\rho }}_{i,j}}$ is greater than unity. As described above, this means that we only present links that are the most distant to the centre, i.e. links with highest scores of being *highly specific*, *highly different* or *highly common*, and are most well classified.

Our first CoDiNA network represents the comparison of the full gene co-expression network of HIV^−^ and HIV^+^ for adults (Fig 5, panel I). In this comparison, CoDiNA was able to identify 80, 509 links and 3, 786 nodes. From those nodes, 455 are common to all network, 172 specific to HIV^−^ and 1, 948 specific to HIV^+^, while the remaining nodes were not classified into any of these categories. When comparing the networks from the children’s data (Fig 5, panel II), CoDiNA identified 243, 645 links and 6, 763 nodes. From those nodes, 573 are common, 3, 546 of specific to HIV^−^ and 926 of specific to HIV^+^, while 1, 718 were unclassified. Our last comparison included data from both, children and adults (Fig 5, panel III). This final CoDiNA network identified 35, 683 links connecting 4, 254 nodes, of which 77 nodes were classified as common. Most links (1, 351 links) in this CoDiNA network are specific to HIV^−^ children, indicating that links are lost due to infection with HIV but also due to development/aging into adults. About three percent (208) links are specific to HIV^+^ children and 11% (430) links to HIV^+^ adults. Only 1.9% (28) percent of links occur in HIV^+^ children and adults. In none of the three networks were any nodes classified as differential nodes, although beta links existed.

**Fig 6:**
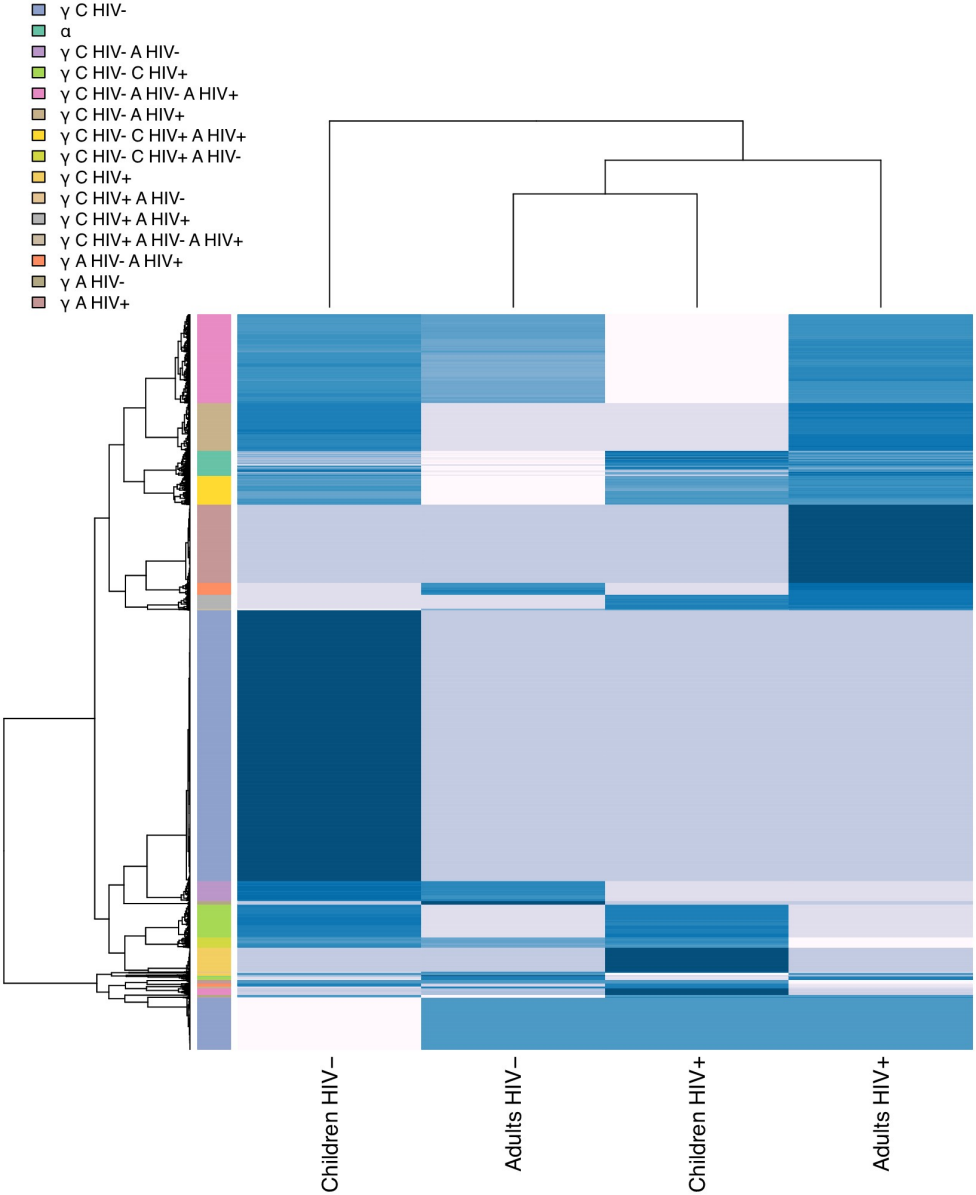
Gene co-expression weighted network heatmap: Heatmap representing the *omega* values of links for each network. The intensity of the color represents the weight of the link. Clusters of links that are stronger or weaker in certain groups of networks can be distinguished. Those clusters coincide with the CoDiNA $\tilde{\Phi }$ subcategories shown on the left.

Looking at the CoDiNA network representations, (Fig 5, right column), nodes belonging to the same CoDiNA $\tilde{\Phi }$ subcategory typically cluster together. When *ω* values of each network are displayed as a heatmap (Fig 6), clusters of links become visible that differ between groups of networks. Those clusters coincide with the CoDiNA $\tilde{\Phi }$ subcategories, demonstrating that CoDiNA captures network differences by comparing multiple networks.

To assess the biological significance of our comparative networks, we next tested for each one of the three CoDiNA networks whether nodes of particular $\tilde{\Phi }$ categories are enriched for genes associated with HIV or TB using an exact Fisher’s test. If p-values were lower than 0.1, we considered it an enrichment. The list of genes associated with HIV Infections, AIDS (Acquired Immunodeficiency Syndrome), sAIDS (Simian Acquired Immunodeficiency Syndrome) or Tuberculosis was retrieved using the tool Gene 2 Disease tool (GS2D) [48]. For our disease enrichment analyses we only considered those genes that were measured in the final CoDiNA networks. Among the unclassified nodes, i.e. the nodes not classified as common, different or specific in the adult CoDiNA network, our enrichment analysis showed an over-representation of TB. This is to be expected since all individuals were infected with TB. In the children CoDiNA network, our enrichment analysis found over-representation of genes related to AIDS and sAIDS for the HIV^+^ children. The HIV^−^ group is enriched for genes related to TB. The CoDiNA network including children and adults contained an enrichment for TB among the unclassified genes, similar to the network for adults. We further found an association to AIDS in HIV^+^ children and sAIDS in adults and children HIV^+^ (Table 2). Thus, CoDiNA was able to successfully identify an enrichment of known genes associated with HIV infections among the specific nodes, providing support for the ability of CoDiNA to retrieve biological meaningful results. Importantly, we were also able to pinpoint sets of genes related to each one of the co-infections.

**Table 2:** Disease Enrichment Analysis for each $\tilde{\Phi }$ category in each CoDiNA network. HIV I: HIV Infections; HIV S: HIV Seropositivity. *p*-values determined by the Fisher’s exact test when testing for enrichment of known disease genes within each category.

Network	Disease	Φ	Known	Observed	*p*-value
Adults	TB	Undefined	25	15	0.13
Adults	HIV I	Common	114	6	0.13
Children	AIDS	Specific to HIV^+^ children	22	8	0.02
Children	sAIDS	Specific to HIV^+^ children	18	7	0.02
Children	TB	Specific to HIV^−^ children	92	49	0.04
Children	HIV I	Common	211	25	0.02
Children	HIV S	Specific to HIV^+^ children	6	3	0.06
Complete	TB	Undefined	42	22	0.01
Complete	AIDS	Specific to HIV^+^ children	10	2	0.09
Complete	sAIDS	Specific to HIV^+^	9	1	0.09

#### CoDiNA suggests new candidate transcription factors as hallmarks of certain cancer types

For the second showcase of our method, we used the gene-expression data from GSE4290 [49], a study of patients with glioma. The dataset contains 157 brain tumor samples of three types (26 astrocytomas, 50 oligodendrogliomas, and 81 glioblastomas). The data was downloaded from the GEO website [42], pre-processed and normalized by ourselves. The cancer expression profiles were normalized with the controls. We used microarray data, which was analyzed using the R environment [50] and the affy [51] package from the Bioconductor. The probe expression levels (RMA expression values) and MAS5 detection *p*-values were computed, and only probesets significantly detected in at least one sample (*p*-value ⩽ 0.05) were considered. After quality control and probe normalization, the probes that were not specific to only one gene were removed. If one gene was bound by more than one probeset, the average expression was computed.

Because TF deregulation is central to disease progression [52, 53] in many disease states, and particularly in cancers, we focused on a comparison of the TF co-expression networks between the three different kinds of tumors. To this end, we calculated the wTO network [54–56] of the TFs for each tumor. We computed the wTO network for each cancer dataset and the controls separately using only the set of 3, 229 unique TF symbols from the Gene Regulatory Factors (GRF)-Catalogue [12, 57], filtered by genes with proteins that also are included in the ENSEMBL protein dataset. For that, wTO R package was used, similarly to the previous example, using the same parameters: Pearson Correlation and a 1000 bootstraps. Links with Benjamini-Hochberg adjusted *p*-values smaller than 0.01 were kept, and links with larger *p*-values were set to zero. We applied CoDiNA to those networks. Within the CoDiNA network, we were able to classify 147 TFs as specific to astrocytomas, 251 specific to oligodendrogliomas and 607 as specific to glioma; only two TFs are specific for both, astrocytomas and gliomas, and six for glioma and oligodendrogliomas; and finally 166 were unclassified. To verify the enrichment of disorders in each one of the $\tilde{\Phi }$ categories, we tested if the number of genes associated to each one of the gliomas under study is different from random expectation using a Fisher’s exact test and the resulting *p*-value was used to filter the results. The association of genes to disorders was retrieved using the tool GS2D [48]. To perform the enrichment test, we considered as background only those genes from GS2D that were expressed in the samples.

In total, the CoDiNA network contains 2, 209 nodes and 206, 856 links with ratios of $\Delta_{i,j}^{*}$ and $\Delta_{{\tilde{\rho }}_{i,j}}$ scores > 1. According to the GS2D tool [48] (weight < 0.10) 51 TFs are described in the literature to be associated to glioblastoma, 8 to astrocytoma, and 3 to oligodendroglioma. In our CoDiNA network, we identified 45 of the 51 known glioblastoma TFs to be associated with glioblastoma (*γ*_*glioblastoma*_ nodes). Further, one of the known astrocytoma TFs and two of the known oligodendroglioma TFs were associated with the respective glioma types by CoDiNA (specific to astrocytoma and specific to oligodendroglioma nodes, respectively), providing strong support for the validity of our comparative network approach.

**Fig 7:**
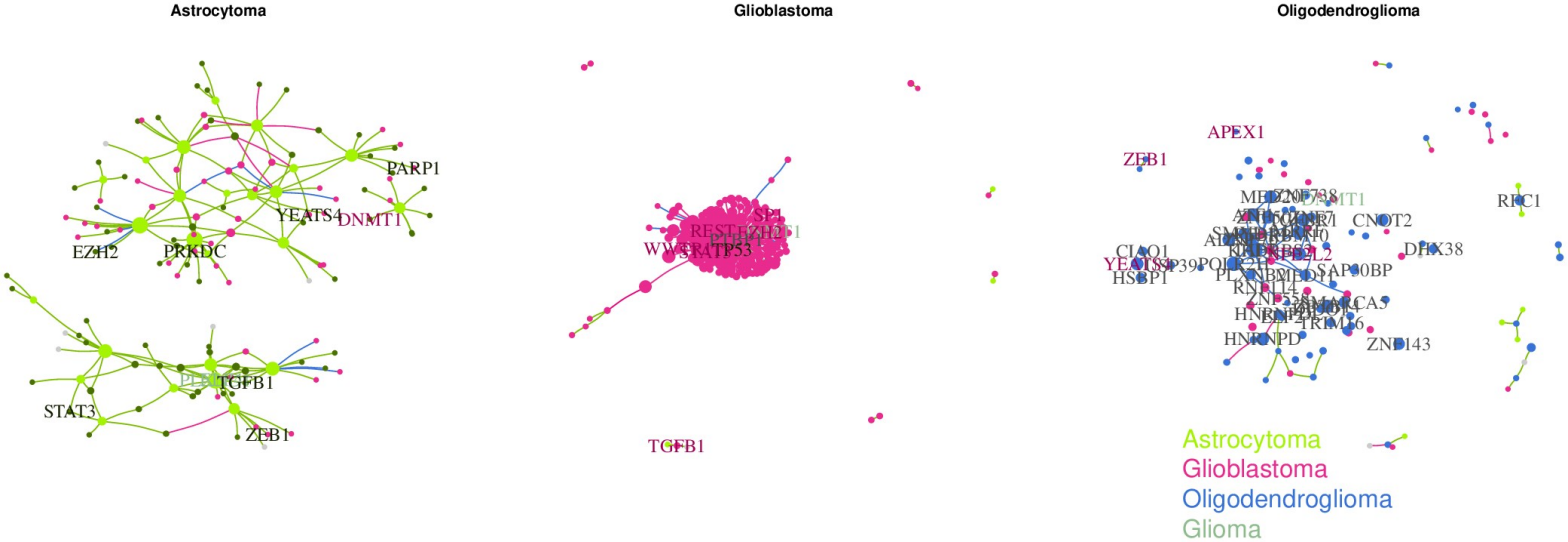
TF-TF CoDiNA networks for each of the Glioma types: CoDiNA identified TFs with specific co-regulatory changes to each cancer, Panel I astrocytoma, Panel II Glioblastoma, Panel III oligodendroglioma. Nodes are coloured according to the type of cancer CoDiNA associated them to. **Panel I**: We can see that mostly glioma and astrocytoma TFs are specific to the astrocytoma network. **Panel II**: The majority of nodes refer to specific changes in co-expression in glioblastoma, but there is an overlap with TFs involved in other gliomas. **Panel III**: Most links and genes are specific to oligodendroglioma, with some overlap of astrocytoma TFs.

In addition, we identified several TFs specifically associated with astrocytoma that were not previously linked to this type of cancer (Fig 7, panel I). The TFs with the 10 strongest associations are: FGD1, TCEAL4, ZNF628, TBPL1, BMP5, MYPOP, HMGA2, PRR3, MIS18BP1, BMP7. Among these, HMGA2, TBPL1, BMP5 and BMP7 were previously described as associated to *neoplasm* and *neoplasm metastasis* [48]. When using a differential gene expression approach (log-linear model; *p*_*adj*_-value < 0.001), we found 4, 444 genes to be differentially expressed in astrocytoma when compared to the controls, of which 670 were coding for TFs. TFs were not enriched among the differentially expressed genes (*p*-value = 0.999, Fisher’s exact test). From the 10 strongest associations, only TBPL1, MYPOP and BMP7 appeared to be differently expressed in astrocytoma, albeit not specific to only astrocytoma.

The most strongly differentiated TFs associated with oligodendroglioma that were not previously described in the literature (Fig 7, panel II) were: SMARCE1, ZNF274, NRG1, ZNF232, UBE2I, TXK, TAF11, PLXNB2, HLX and SAP30BP. Of these, SMARCE1, NRG1, PLXNB2 and UBE2I were previously described as associated to *neoplasm invasiveness* and *neoplasic cellular transformation* [48]. Similarly, using a differential gene expression approach, we identified 5228 differentially expressed genes in oligodendroglioma when compared to the controls, including 852 TFs. A significant enrichment for TFs was found (*p*-value = 0.003, Fisher’s exact test). From our top 10 specific TFs, only 3 are differently expressed in this cancer type (PLXNB2, NRG1, SAP30BP). As in the astrocytoma case, these 3 genes were not specifically differentially expressed only in oligodendroglioma.

The ten TFs most specific for glioblastoma according to CoDiNA analysis (but not previously described) (Fig 7, panel III) were: ZNF558, PTBP1, XRN2, RNF114, ZNF45, ZNRD1, KHDRBS2, RFXANK, NIFK and ZNF540. Here, the genes PTBP1, RNF114, XRN2 and ZNRD1 are described as associated with other *neoplasms* [48]. 8204 genes including 1288 showed differential expression in glioblastoma, when compared to controls. We found a significant enrichment for TFs (*p*-value = 0.032, Fisher’s exact test) among differentially expressed genes. All ten TFs specific to glioblastoma were found to be differentially expressed in all three cancers.

Taken together, this suggests, that CoDiNA can be applied to detect novel candidate genes for specific cancer types, which would have been missed if only differential gene expression was analyzed (i.e., from our top ten candidates, only 3 are differentially expressed). We were able to identify TFs as specific nodes that had previously been associated with other neoplams, but not the types of glioma under study, in important roles in the differential glioma network, indicating that those TFs are also deregulated in those disorders. Importantly, if only differential gene expression had been analyzed, many of these new candidates would have been overlooked or not been found to be specifically associated with a particular type of cancer.

## Comparing CoDiNA to other methods

For a comparison of CoDiNA to other methods we need to consider at least two main aspects. First, do the methods solely perform a network comparison or do they also construct the networks? DiffCorr, DCEA, DCGL, CSD and CoXpress construct the networks prior comparison using their own methods. Thus, comparing their results to CoDiNA would not allow us to disentangle whether differences are due to network construction or the method of network comparison. Second, which network sizes can be compared with a given method? To the best of our knowledge, CoDiNA is the only method that allows for networks of the size of whole vertebrate transcriptomes. CompNet, ConMod, and ModMap enable a comparison of small and medium size networks. We are thus restricted to using the glioma dataset instead of the HIV dataset for a method comparison. Because ModMap is still under development and ConMod is suggested for finding conserved clusters from a much larger set of networks than only three, we compare CoDiNA with CompNet.

While CoDiNA takes into account the weight of a link for its classification, CompNet uses unweighted networks. We thus converted our weighted wTO networks into binary networks. While CoDiNA outputs links and nodes that are common, specific or different for each network together with a p-value, CompNet identifies the union, intersection and exclusive links among a selected set of networks, and outputs network communities and network properties such as centrality measures (betweenness and closeness) along with node degree. CompNet, opposed to CoDiNA, does not report a mutually exclusive category for each node or link. Instead it defines three networks: the union, that contains all nodes and links, the exclusive, that contains sub-networks that are not present in all networks and finally, the intersection, that are sub-networks that exist in all networks simultaneously. None of the categories are comparable to CoDiNA’s Φ or $\tilde{\Phi }$. CompNet allows selecting nodes based on betweenness and degrees as the best candidates for each category.

CompNet was able to classify 1220 nodes and 80838 links as being in the union of the networks, 290 nodes with 7937 links in the intersection, and 1219 nodes and 80845 links that are exclusive. As mentioned above, 51 TFs are associated to glioblastoma, 8 to astrocytoma, and 3 to oligodendroglioma according to the GS2D tool. Among the 290 TFs in the intersection network, no TF was related to oligodendroglioma, and 1 and 9 TFs were related to astrocytoma and glioblastoma, respectively. Among the exclusive nodes, GS2D found 26 TFs to be related to glioblastoma, 2 TFs related to astrocytoma, and 1 related to oligodendroglioma. In contrast, CoDiNA had recovered 45 TFs related to glioblastoma, 1 to astrocytoma, and 2 to oligodendroglioma among the specific nodes. While CompNet and CoDiNA identified about the same number of TFs related to the respective cancer type in their exclusive/specific TF set, CoDiNA found almost the double number of TFs to be specific to glioblastoma.

Since CompNet does not provide p-values, we selected its top 10 TFs with the highest betweenness and the highest degree as was done in the original CompNet publication for disease enrichment analysis. According to the GS2D tool, among genes with highest degree in the intersection network 2 TFs, FOXM1 and PRKDC, are associated to glioblastoma. PRKDC is also among the TFs with highest betweenness. Surprisingly, none of the genes with highest degree or betweenness in any of the exclusive networks was associated with any of the investigated tumors. We conclude that CoDiNA has better performance in identifying disease-associated TFs in this study.

## A package to compare multiple networks

**Fig 8:**
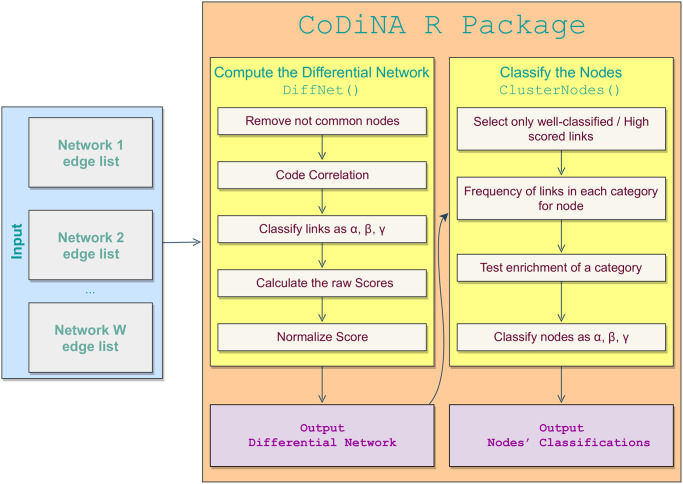
Workflow process of the CoDiNA R package. Input data for the CoDiNA R package can be any networks, filtered for containing only significant links (according to the network construction method used). Edge list is a list containing all the links and their weights. The user can assign a weight of zero to links for which the *p*-value is not significant. The function MakeDiffNet() classifies the links into the Φ and $\tilde{\Phi }$ categories, calculates and normalises the scores. Its output is used as input for assigning the nodes into categories by the function ClusterNodes(). The plot() function can be used on the output from MakeDiffNet() and automatically calls the function ClusterNodes().

To make the proposed methodology publicly available, we developed an R package, called CoDiNA, in which all the presented steps are implemented. The R package also includes an interactive visualisation tool. CoDiNA’s workflow analysis is presented in Fig 8. The functions included in the package are:

- normalize(): Normalizes a variable according to Eq (4);
- OrderNames(): Reorder the names of the nodes for each link in alphabetical order;
- MakeDiffNet(): Categorize all the links into Φ, $\tilde{\Phi }$ and the combination of signs a link has across all compared networks. It also computes the normalized scores;
- plot: Classifies the nodes into Φ and $\tilde{\Phi }$ following a user-defined cutoff for the chosen distance and plots the network in an interactive graph, where nodes and links can be dragged, clicked and chosen according to their categories. The size of a node is relative to its degree. Nodes and links that belong to the *α* (*common*) group are colored in shades of green; Nodes belonging to the *β* (*different*) group are colored in shades of red; Nodes of the *γ* (*specific*) group are colored in shades of blue. Nodes are categorized according to Φ and $\tilde{\Phi }$ according to a *χ*^2^ goodness-of-fit test as defined above. If a node is group-undetermined it is colored in grey. The user can also choose a layout for the network visualization from those available in the igraph package [58]. It is further possible to cluster nodes, using the parameter MakeGroups, and the user may select among the following clustering algorithms: “walktrap” [59], “optimal” [60], “spinglass” [61–63], “edge.betweenness” [64, 65], “fast_greedy” [66], “infomap” [67, 68], “louvain” [69], “label_prop” [70] and “leading_eigen” [71].

The CoDiNA package also contains three datasets for illustrative purposes.

- The AST data.table contains the nodes and the wTO of TFs, from GSE4290 [49] for astrocytomas;
- The GLI data.table contains the nodes and the wTO of TFs, from GSE4290 [49] for glioblastomas;
- The OLI data.table contains the nodes and the wTO of TFs, from GSE4290 [49] for oligodendrogliomas;
- And the CTR data.table contains the nodes and the wTO of TFs, from GSE4290 [49] for controls.

CoDiNA is open source and freely available from CRAN at https://cran.r-project.org/web/packages/CoDiNA/ under the GPL-2 Open Source License, and is platform independent.

## Conclusion

We presented a novel method, CoDiNA, that allows for the systematic comparison of multi-dimensional data across more than two conditions and the representation of the results as a single network. While other methods perform the comparison at the level of modules, our method identifies links and nodes that are common to all networks under consideration, specific to at least one network, or have different signs among the compared networks. The conceptual differences between CoDiNA and other methods do not allow for a performance comparison between them apart from CompNet, where CoDiNA was able to identify almost twice as many specific TFs for glioma. However, the researcher should decide if a comparison at the level of modules or links and nodes is of interest in the study or use both types of analyses complementary.

Important to note is that coexpression networks often suffer from a high false positive rate for inferred links, which can confound a comparison at the level of individual links. We thus recommend to construct the networks with a method that reduces false positives. For instance, weighted topological overlap networks [9, 11, 72] can be calculated from coexpression matrices to focus on commonalities in coexpression patterns instead of on individual coexpressions, thus greatly reducing false positive inferences. The user can further define a cutoff based on weights or p-values to remove unreliable links [8]. Then, wTO networks can be used as inputs for CoDiNA. Another measure to reduce false positives is to use Consensus Networks calculated from the integration of several networks retrieved from similar conditions, to include only links that have been repeatedly observed across biological repeats [8]. CoDiNA can then perform the comparison of Consensus Networks from different conditions. Most other co-expression comparison methods rely on expression data as input and construct their networks based on pre-defined methods, preventing the researcher from comparing networks constructed with a method of her choice.

A further limiting factor of other methods is the size of the networks that can be compared. While CompNet, a GUI based tool, can compare two or more networks and take as input user-constructed networks, it is only able to handle small or medium sized networks. CoDiNA instead can analyze whole transcriptome networks within minutes on a conventional laptop.

To leverage biological information onto the differential network analysis, we recommend performing downstream analyses after defining common, differential and specific nodes, such as GO or disease enrichment tests for each node category. This might be more important for differential and specific nodes, as it should be unlikely that the same links exist by chance across multiple conditions.

To evaluate CoDiNA, we applied it to a neurogenesis study and identified genes involved in neuronal differentiation. One of these genes was experimentally modified, and its impact during neurogenesis was verified. Applying our method to HIV datasets, CoDiNA retrieved networks with HIV specific links, whose nodes were enriched for genes associated to HIV, AIDS, or sAIDS. We further identified network signatures that are specific to any of three types of glioma. This suggests that our method produces biologically meaningful results that go beyond describing gene expression differences. More importantly, we were able to propose new candidate genes as associated with particular phenotypes or disorders and their interactions.

We expect that our method will be helpful for many diverse studies comparing network data generated from multiple conditions, such as different diseases, tissues, species or experimental treatments. If desired, similar conditions could also be grouped first at the level of Consensus Networks before comparing them with CoDiNA. This might be useful in cases such as chronic kidney diseases or Alzheimer’s disease, where some stages might not be easy to distinguish from each other. Furthermore, CoDiNA is not limited to the analysis of co-expression networks, but can be applied to comparing any type of network.

## Materials and methods

### Overview of the proposed method

We first introduce the idea behind CoDiNA briefly and will in the next section present the algorithm and the method in details.

### The CoDiNA method

Let $W=\{w_{1},\ldots ,w_{W}\}$ be a set of *W* **independent** networks (objects composed by nodes and links). Each element of W is defined as a graph $G(N_{k},L_{k},\rho_{k})$, where $N_{k}=\left\{n_{k,1},\ldots ,n_{k,N}\right\}$ is the *N*-dimensional set of nodes of the *k*-th network, being the node indices expressed by *i* or *j*. Besides, $L_{k}=\left\{\ell_{k,1},\ldots ,\ell_{k,L}\right\}$ denotes the set of links identified from nodes *i* and *j*, and ***ρ***_*k*_ is a vector containing specific link weights (*ρ*_*i*,*j*,*k*_ ∈ [−1, 1]), relative to the connection between nodes *i* and *j* in the network *k*.

#### Considerations on missing nodes

It is possible, that not all genes have been measured in all conditions for which the networks are to be compared, which could produce a problem of missing nodes in the comparison. There are several possible strategies for dealing with missing nodes (e.g., [38]). We keep all genes that were measured in all experiments for further analyses. However, if measurement data does not exist, the node for that gene cannot be included in the network comparison. For example, when comparing three networks, Gene A’s correlations were measured in two out three conditions. Thus, it is not possible to infer that Gene A is not correlated with any genes in the third condition; the information is simply missing. This situation is to be distinguished from the case where Gene A was measured in all conditions, but has no significant correlation in one of the networks. To prevent the erroneous inference that a particular node is associated with a specific condition, when in fact that *specific* node was not measured in the other conditions, we remove nodes that have not been measured in all conditions (Algorithm 1). Subsequently, all links of a node not present in all networks will be removed from the networks in which it is present. Importantly, our node removal does not exclude genes that were not expressed in a given condition (e.g., genes with expression values of zero or below a user defined threshold). Such genes will stay in the analysis with an expression value of zero. We are also not removing nodes without any significant links. The user should define a threshold for keeping links, for instance based on having a predefined *p*-value for the correlations, and links not reaching that threshold should be assigned a weight of zero. This step permits that all genes that were **measured** stay in the comparison. It is a different issue, if the user knows that certain genes only exist in a specific condition, e.g., if a gene was knocked-out, or if it is species specific. In such a case, the integration of condition specific genes into the network can be analyzed by keeping it with its links in the network(s) in which it exists and artificially adding it to the network(s) in which it does not exist. In the network(s) in which it is not present, all its interactions need to be assigned a weight to zero. This way, significant links in the networks of the conditions in which the gene exists, will show up as species specific or condition specific in the CoDiNA network.

**Algorithm 1** Description of the RemoveNodes procedure

**Input**: Set of $\left\{N_{1},\ldots ,N_{W}\right\}$ nodes that belongs to the networks contained in W

**Output**: Set of common nodes to all *W* networks

1: **procedure** RemoveNodes $N_{1},\ldots ,N_{W}$

2:  Set_Nodes = ${⋂}_{k=1}^{W}N_{k}$

3:  **return** Set_Nodes.

4: **end procedure**

#### Categorization of links

Next, the link weight in each network is categorized. By default, the interval for the links’ weights is partitioned into three equal parts (*τ* = 1/3), which will be denoted as corresponding to a positive link, negative link or neutral link (Algorithm 2). When the interest does not lie in characterizing specific links, *τ* can be set to zero. Moreover, it is important to normalize the networks before deciding on the *τ* threshold. Each link is categorized as

$$\begin{array}{cc} {\tilde{\rho }}_{i,j,k}= & \left\{ \begin{array}{cc} -1 & \text{if}\;\rho_{i,j,k}<-\tau \\ 1 & \text{if}\;\rho_{i,j,k}>\tau \\ 0 & \text{otherwise}, \end{array}\right. \end{array}$$

where ${\tilde{\rho }}_{i,j,k}$ is an integer transformation of the link weight based on the threshold *τ*. If a particular link categorical weight ${\tilde{\rho }}_{i,j,k}$ is zero in all *W* networks, this link is removed from posterior analyses.

If the compared networks have different link-weight ranges, these may be normalized by using a multiplicative (*stretch*) parameter. This parameter forces the *ρ*_*i*,*j*,*k*_ values to lie within [−1, 1]. This is particularly important for comparing networks constructed from different and not directly comparable measures. This parameter is also valid for networks constructed with methods producing only links with weights greater than zero. In these cases, weights will be stretched within the unit interval.

**Algorithm 2** Description of the links categorization algorithm

**Input**: Set of *W* networks with $\left\{N_{1},\ldots ,N_{W}\right\}$ nodes (*N* ⩾ 2;*W* ⩾ 2)

**Output**: Links weight categorized into −1, 0 or 1

1: Set *τ* ⩾ 0;

2: By default *τ* ← 1/3;

3: **procedure** AssignClasses

4:  **for** *i* ← 1 **to** *N* **do**

5:   **for** *j* ← 1 **to** *N* **do**

6:    **for** *k* ← 1 **to** *W* **do**

7:     **if** *ρ*_*i*,*j*,*k*_ < *τ* **then**

8:      ${\tilde{\rho }}_{i,j}\leftarrow -1;$

9:     **else if** *j* > *τ* **then**

10:      ${\tilde{\rho }}_{i,j}\leftarrow 1;$

11:     **else**

12:      ${\tilde{\rho }}_{i,j}\leftarrow 0;$

13:     **end if**

14:    **end for**

15:   **end for**

16:  **end for**

17: **end procedure**

After the correlation values are coded into the categorical variables ${\tilde{\rho }}_{i,j}$, each link is assigned to an additional category, called Φ, describing its conservation across the compared networks. Φ is a variable that takes three categorical values: *α*, *β* and *γ*. This classification approach assigns an *α*, *β* or *γ* to each of the links by defining Φ as

$$\begin{array}{cc} \Phi_{i,j}= & \left\{ \begin{array}{cc} \alpha & \text{if}(\sum_{k=1}^{W}|{\tilde{\rho }}_{i,j,k}|=W)\;\;\text{and}\;\;(|\sum_{k=1}^{W}{\tilde{\rho }}_{i,j,k}|=W)\\ \beta & \text{if}(\sum_{k=1}^{W}|{\tilde{\rho }}_{i,j,k}|=W)\;\;\text{and}\;\;(|\sum_{k=1}^{W}{\tilde{\rho }}_{i,j,k}|<W)\\ \gamma & \text{otherwise}, \end{array}\right. \end{array}$$ (1)

where *α* is assigned to links that exist in all categories with the same sign, meaning that their co-expression pattern does not change depending on the condition. Those links are probably more robust and would have higher costs to be changed. The categorical value *β* is assigned to links that exist in all networks, however, with different co-expression sign in at least one network. This indicates that the regulatory pattern in that network has changed. Finally, *γ* is assigned to links that exist only in a subset of networks. These links represent rewiring events in the co-expression patterns that are condition specific. Algorithm 3 describes this categorization process.

Because it is impossible to infer which network has changed or specific links based on the Φ category alone, we also introduce a $\tilde{\Phi }$ subcategory. This classification step is particularly important for links that are classified as *β* or *γ* type, to indicate, in which network(s) the link is different or specific.

**Algorithm 3** Description of the Φ algorithm

**Input**: Set of *W* networks with $\left\{N_{1},\ldots ,N_{W}\right\}$ nodes (*N* ⩾ 2;*W* ⩾ 2)

**Output**: Network with links categorized into *α*, *β* or *γ*

1: Set *τ* ⩾ 0;

2: **procedure** PhiLinks

3:  **for** *i* ← 1 **to** *N* **do**

4:   **for** *j* ← 1 **to** *N* **do**

5:    **for** *k* ← 1 **to** *W* **do**

6:     **if** $\sum_{k=1}^{W}|{\tilde{\rho }}_{i,j,k}|=0$ **then**

7:      remove link;

8:     **else if** $(\sum_{k=1}^{W}|{\tilde{\rho }}_{i,j,k}\left|=W)\;\;\text{and}\;\;(\right|\sum_{k=1}^{W}{\tilde{\rho }}_{i,j,k}\left|=W\right)$ **then**

9:      Φ_*i*,*j*_ ← *α*;

10:     **else if** $(\sum_{k=1}^{W}|{\tilde{\rho }}_{i,j,k}\left|=W)\;\;\text{and}\;\;(\right|\sum_{k=1}^{W}{\tilde{\rho }}_{i,j,k}\left|<W\right)$ **then**

11:      Φ_*i*,*j*_ ← *β*;

12:     **else**

13:      Φ_*i*,*j*_ ← *γ*;

14:     **end if**

15:     Calculate the Euclidean Distance Δ_*i*,*j*_ (Eq 2)

16:     Calculate the penalized Euclidean Distance $\Delta_{i,j}^{*}$ (Eq 3)

17:     Calculate the normalized penalized Euclidean Distance $\Delta_{i,j}^{**}$ (Eq 4).

18:    **end for**

19:   **end for**

20:  **end for**

21: **end procedure**

To illustrate the concept of subcategory, assume the following ${\tilde{\rho }}_{i,j}$ of a particular link in 3 networks: ${\tilde{\rho }}_{i,j,A}=1$; ${\tilde{\rho }}_{i,j,B}=1$ and ${\tilde{\rho }}_{i,j,C}=1$. Because the value 1 is common in the three networks, this Φ category is clearly *α*, and no further explanation is needed. Now, take as a second example, ${\tilde{\rho }}_{i,j,A}=1$; ${\tilde{\rho }}_{i,j,B}=-1$ and ${\tilde{\rho }}_{i,j,C}=1$. Its Φ class is *β*, but this classification does not indicate, in which network the change occurs. This information is stored in the $\tilde{\Phi }$. In this example, its $\tilde{\Phi }$ class is *β*_*B*_. As a final example, assume that the ${\tilde{\rho }}_{i,j}$ weight of the three networks are 0, 1 and 1 for Networks A, B and C, respectively. This link does not occur in network A, so it is a *γ* link, that is specific to networks *B* and *C*. Therefore, its $\tilde{\Phi }$ category is *γ*_*B*.*C*_. According to this concept, each link receives a subcategory, $\tilde{\Phi }$, based on the pattern of networks in which that link exists. Finally, we note, which sign a link has in each networks. The maximum number of distinct combinations of signs is (3^*W*^ − 1). The combination for which all categorical values are equal to zero is removed from analyses. Important to note is that our method assumes the first network to be the reference network. The reference network acts as baseline or control to which all links are compared to. If the reference network is changed, the notations of the *beta* and *gamma* links and nodes will also change. For example, if “controls” are chosen as the reference, the $\tilde{\Phi }$ categories will be called *β*_*cases*_ and *γ*_*cases*_. If “cases” are defined as reference, the outcome will be *β*_*controls*_ and *γ*_*controls*_, respectively. The interpretation of the results, however, does not change.

When all links are assigned to a Φ category and further subcategorised as $\tilde{\Phi }$, it is necessary to score them to identify those that are stronger. For every link *ρ*_*i*,*j*,*k*_, we interpret the array of link weights (*ρ*_*i*,*j*,1_, …, *ρ*_*i*,*j*,*W*_) as a point in a *W*-dimensional Euclidean space. In particular, as each link weight is bounded, all points are contained in the cube determined by the Cartesian product [−1, 1]^*W*^. A link that is closer to the center of the *W*-dimensional cube is weaker than a link closer to the cube’s edges. Based on that, the Euclidean distance to the origin of the space (Fig 3a) is calculated for all links as

$$\Delta_{i,j}=\sqrt{\sum_{k=1}^{W}\rho_{i,j,k}^{2}}.$$ (2)

However, since links closer to corners will trivially have a larger Δ_*i*,*j*_ compared to the others, all distances are penalized by the maximum theoretical distance a link can assume in its category. Consequently, we define a penalized distance as

$$\begin{array}{c} \Delta_{i,j}^{*}=\sqrt{\frac{\sum_{k=1}^{W}\rho_{i,j,k}^{2}}{\sum_{k=1}^{W}\left|{\tilde{\rho }}_{i,j,k}\right|}}, \end{array}$$ (3)

which lies in the unit interval.

We also suggest a second step to normalize the resulting values in each Φ and $\tilde{\Phi }$ categories to overcome the challenge that some categories have more links than others. This measure is defined as

$$\begin{array}{c} \Delta_{i,j}^{**}=\frac{\Delta_{i,j}^{*}-\min\left\{\Delta^{*}\right\}}{\max\left\{\Delta^{*}\right\}-\min\left\{\Delta^{*}\right\}}, \end{array}$$ (4)

where Δ* is a vector containing all $\Delta_{i,j}^{*}$ values.

Two different approaches may be applied to this normalization:

- Normalize all the links independently of its Φ and $\tilde{\Phi }$ categories: Here, it is not considered if links belonging to the same Φ are situated near the surface or closer to the center of the cube;
- Normalize links according to their Φ and $\tilde{\Phi }$ class: In this alternative, all the categories are a part of the final output. This means that if all links from one of the Φ categories are closer to the cube’s center compared to the links of the other Φ categories, they will be displayed in the final network. Therefore, all Φ and $\tilde{\Phi }$ links have the same chance of being included in the network.

Another important score calculated by CoDiNA is called the *Internal Score* (Fig 3b), denoted by $\Delta_{{\tilde{\rho }}_{i,j}}$. It measures the distance from the link weights *ρ*_*i*,*j*,*k*_ to their categorical weights ${\tilde{\rho }}_{i,j,k}$. In other words, in a 3-networks comparison, if a link is considered an *α* with positive links in all networks (1, 1, 1), we calculate its distance to the point (1, 1, 1). A *β* link that has a $\tilde{\rho }$ of (1, 1, −1) has its distance calculated to the point (1, 1, −1). And for a *γ* link with $\tilde{\rho }$ of (0, 1, 1) the distance is calculated to (0, 1, 1). This score allows us to identify links that are most well classified into a particular $\tilde{\Phi }$ category.

Because the two scores $\Delta_{i,j}^{**}$ and $\Delta_{{\tilde{\rho }}_{i,j}}$ are highly negatively correlated (not linearly), their ratio can be used to describe how well a link is classified for a specific category. Links with high $\Delta_{i,j}^{**}$ but low $\Delta_{{\tilde{\rho }}_{i,j}}$ are strong and well-classified for their respective category. This conclusion can be reached straightforward: if a link is very well classified, its *ρ*_*i*,*j*,*k*_ is close to ${\tilde{\rho }}_{i,j}$, therefore its distance is close to zero. If a link has high *ρ*_*i*,*j*,*k*_ its distance to the central point of the n dimensional is close to one. For a well defined unstretched CoDiNA network, this ratio should be greater or equal than 1.

#### Categorization of nodes

To better describe network differences and their potential functional consequences, we also classify the nodes. To define the Φ category of a particular node, we make a frequency table of how many times each node had a link in each Φ category and $\tilde{\Phi }$ subcategory. Using a *χ*^2^ goodness-of-fit test, we test the hypothesis that the links of a node are distributed equally in all categories tested. This is done for each node to test if the frequency of its links in each Φ and $\tilde{\Phi }$ categories is different than expected by chance (Fig 3d). If the null hypothesis is rejected, the Φ-category with the maximum number of links is assigned to that particular node. Similarly, the same is done for the $\tilde{\Phi }$ (Algorithm 4). When a tie between two categories exists, we are unable to classify a node into a category, and thus declare it to be undefined.

**Algorithm 4** Description of the node-categorization algorithm

**Input**: Set of $\left\{N_{1},\ldots ,N_{W}\right\}$ nodes (*N* ⩾ 2)

**Output**: Node classified as *α*, *β* or *γ* type

1: **procedure** PhiNodes

2:  **for** *i* ← 1 **to** *N* **do**

3:   Φ_*α*_ ← # *α*;

4:   Φ_*β*_ ← # *β*;

5:   Φ_*γ*_ ← # *γ*;

6:   Test if Φ_*α*_ ≠ Φ_*β*_ ≠ Φ_*γ*_

7:   **if** Φ_*α*_ **then**

8:    Φ_*i*,*j*_ ← *α*

9:   **else if** Φ_*β*_ **then**

10:    Φ_*i*,*j*_ ← *β*

11:   **else if**

12:    Φ_*i*,*j*_ ← *γ*

13:   **else**

14:    Φ_*i*,*j*_ ← Undefined

15:   **end if**

16:  **end for**

17: **end procedure**

Algorithm 5 shows the complete pseudo-code for the CoDiNA method.

**Algorithm 5** Description of the CoDiNA algorithm

1: Call: RemoveNodes

2: Call: AssignClasses

3: Call: PhiLinks

4: Call: PhiNodes
